# Supplementary material for: Lactate Activates AMPK Remodeling of the Cellular Metabolic Profile and Promotes the Proliferation and Differentiation of C2C12 Myoblasts
Source: Int J Mol Sci. 2022 Nov 13;23(22):13996. doi: 10.3390/ijms232213996 (PMC9694550; doi:10.3390/ijms232213996)
Supplement: Supplementary file 1 [file ijms-23-13996-s001.zip › ijms-1983946-supplementary.pdf]

Supplementary Materials

Lactate Activates AMPK Remodeling of the Cellular Metabolic Profile and Promotes the Proliferation and Differentiation of C2C12 Myoblasts

Yu Zhou [1,†], Xi Liu [1,†], Caihua Huang [2] and Donghai Lin [1,*]

[1] Key Laboratory for Chemical Biology of Fujian Province, MOE Key Laboratory of Spectrochemical Analysis and Instrumentation, College of Chemistry and Chemical Engineering, Xiamen University
[2] Research and Communication Center of Exercise and Health, Xiamen University of Technology, Xiamen 361024, China
* Correspondence: dhlin@xmu.edu.cn
† These authors contributed equally to this work.

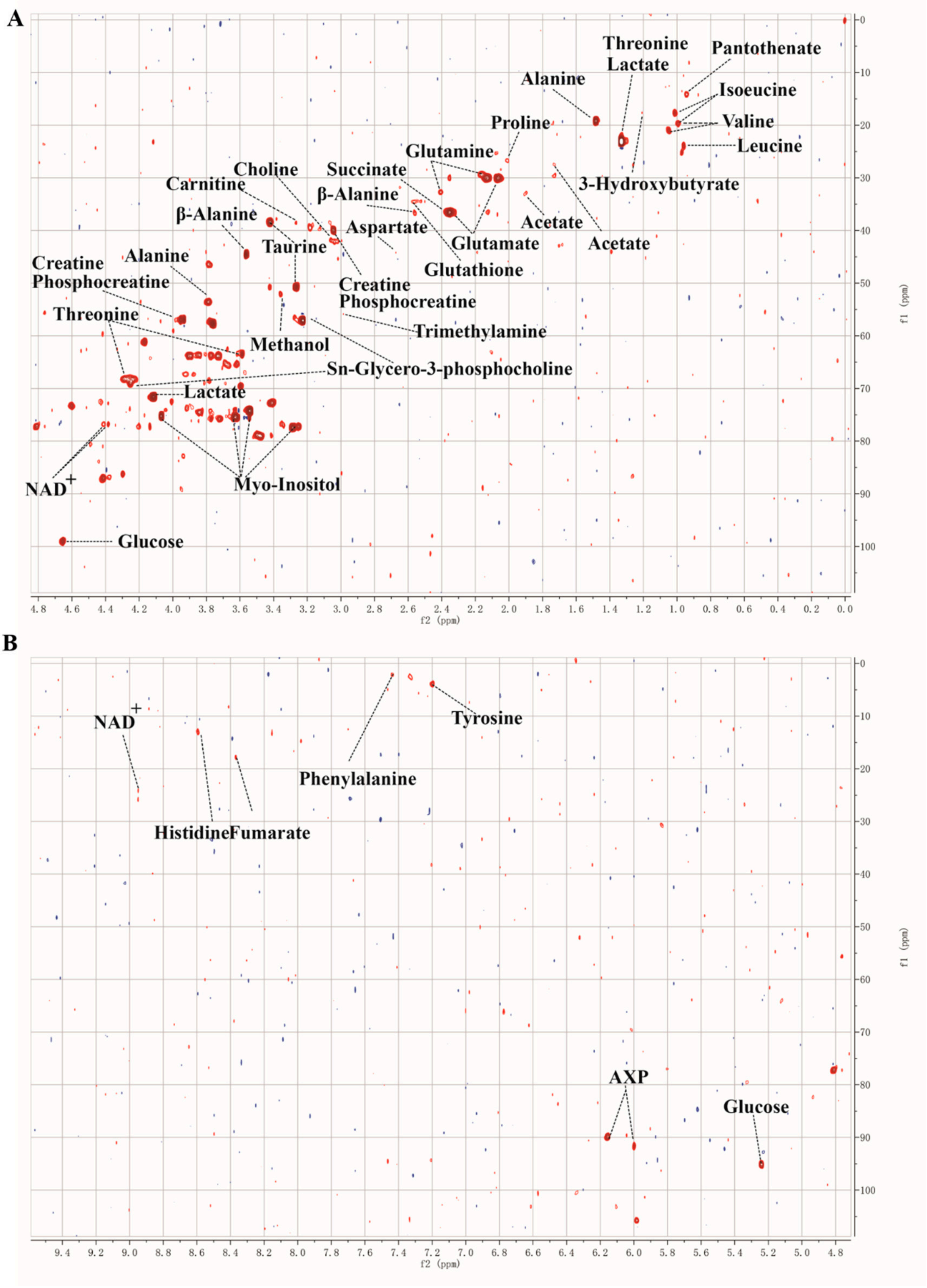

Figure S1. Representative 2D ^{1}H-^{13}C HSQC spectra of aqueous extracts derived from C2C12 myoblasts recorded on an 850 MHz NMR spectrometer. Selected regions of 0.0-4.8 ppm (A), 4.8-9.6 ppm (B).

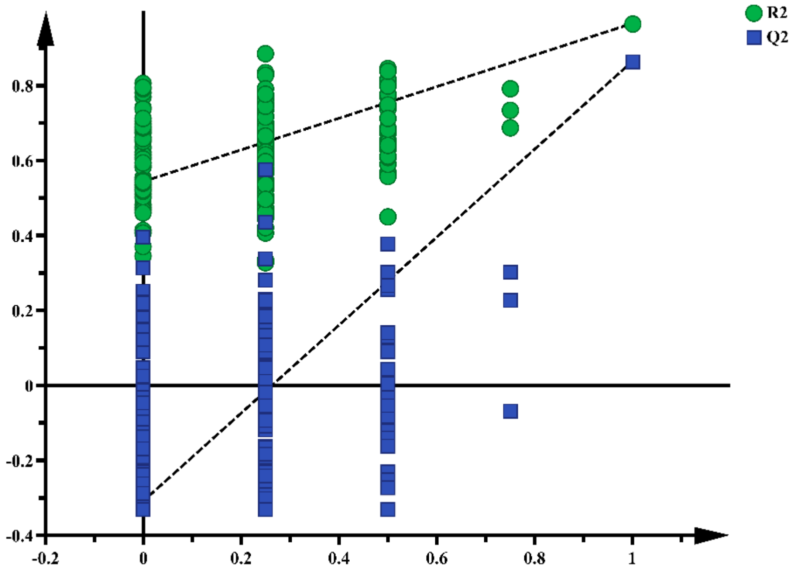

Figure S2. Permutation test of the PLS-DA model for the 1D ^{1}H-NMR spectra recorded from aqueous extracts derived from C2C12 myoblasts with or without lactate supplementation (n=200).

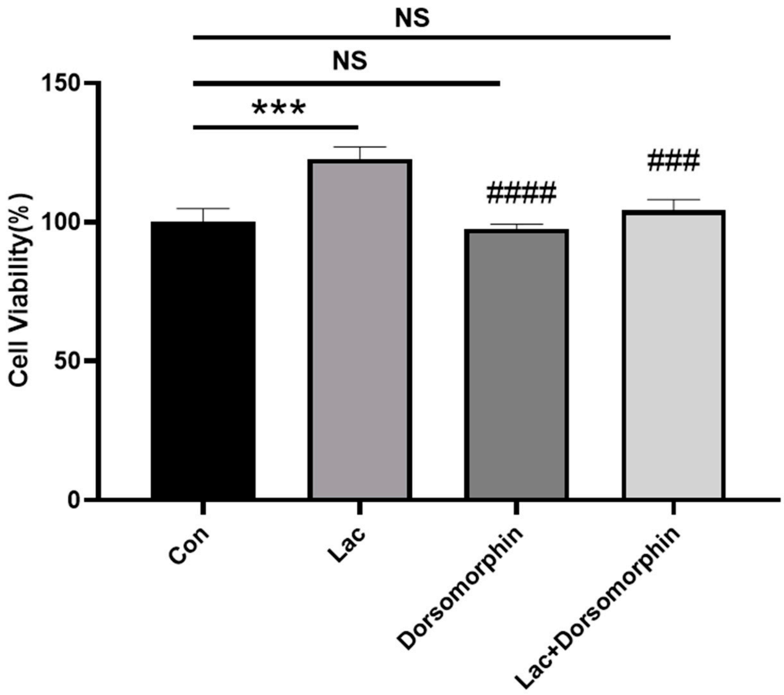

Figure S3. Viabilities of C2C12 cells with different treatments (lactate, dorsomorphin and lac+dorsomorphin) relative to controls measured by the MTS assay (n = 10). *: Con *vs.* Lac, #: *vs.* Lac. $p < 0.001$ (***), and $p < 0.001$ (###), $p < 0.0001$ (####), $p > 0.05$ (NS).

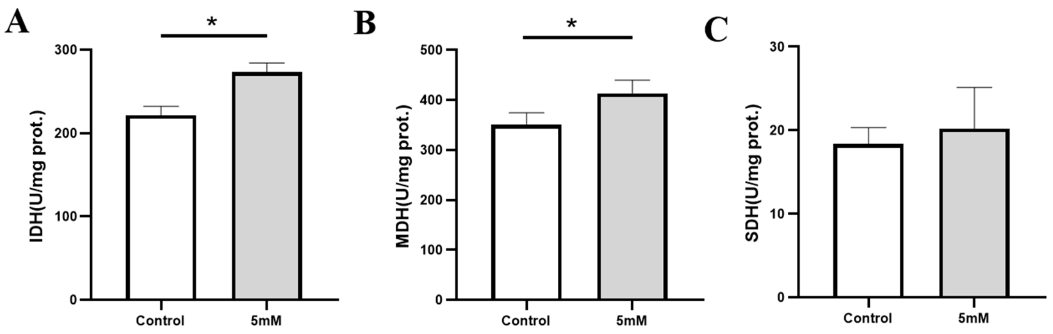

Figure S4. Enzymatic activities of three metabolic enzymes in the TCA cycle with and without 5 mM lactate supplementation. (A) isocitrate dehydrogenase (IDH); (B) malate dehydrogenase (MDH); and (C) succinate dehydrogenase (SDH). n=5 for each group. *p* < 0.05 (*).

Table S1. Resonance assignments of metabolites in 1D ^{1}H-NMR Spectra of C2C12 myoblasts

NO	Metabolites	δ ^{1}H (ppm) and multiplicity	Moieties
1	Pantothenate	0.88(s), 0.92(s)	CH_3, CH_3
2	Leucine	0.96(d), 0.97(d), 1.69(m), 1.70(m), 1.73(m), 3.73(m)	α-CH_3, α-CH_3, γ-CH, β-CH_2, α-CH
3	Isoleucine	0.94(t), 1.01(d), 1.21(m), 1.42(m), 2.00(m), 3.67(d)	δ-CH_3, γ-CH_3, half γ-CH_2, half γ-CH_2, β-CH, α-CH
4	Valine	0.99(d), 1.05(d), 2.26(m), 3.60(d)	γ-CH_3, γ-CH_3, β-CH, α-CH
5	Ethanol	1.17(t), 3.65(q)	δ-CH_3, CH_2
6	3-Hydroxybutyrate	1.20(d), 2.30(q), 2.39(q), 4.14(m)	γ-CH_3, β-CH_2, γ-CH
7	Alanine	1.47(d), 3.78(q)	β-CH_3, α-CH
8	Acetate	1.91(s)	CH_3
9	Proline	1.99(m)	γ-CH_2
10	Glutamate	2.08(m), 2.12(m), 2.34(m), 2.37(m), 3.75(m)	half β-CH_2, half β-CH_2, half γ-CH_2, half γ-CH_2, α-CH
11	Succinate	2.41(s)	CH
12	Glutamine	2.13(m), 2.45(m), 3.77(t)	γ-CH_2, β-CH_2, α-CH
13	Methionine	1.98(m), 2.13(s), 2.17(m), 2.66(dd), 3.78(m)	δ-CH_3, γ-CH_2, β-CH_2
14	Aspartate	2.68(dd), 2.81(dd), 3.90(dd)	β-CH_2, α-CH
15	Asparagine	2.84(dd), 2.94(dd), 4.00(dd)	half β-CH, half β-CH, α-CH
16	Trimethylamine	2.88(s)	CH_3
17	Glutathione	2.15(m), 2.55(m), 2.96(m), 3.77(m), 4.56(m)	β-CH_2, γ-CH_2, CH_2-SH, α-CH&CH_2-NH, CH-NH
18	Lysine	1.43(m), 1.50(m), 1.73(m), 1.89(m), 1.92(m), 3.02(t), 3.75(t)	γ-CH_2, half γ-CH_2, δ-CH_2, β-CH_2, ε-CH_2, α-CH
19	Creatine	3.04(s), 3.93(s)	N-CH_3, α-CH_2

20	Phosphocreatine	3.05(s), 4.05(s)	$N\text{-}CH_3$, CH_2
21	β-Alanine	2.54(t), 3.17(t)	CH_2, CH_2
22	Choline	3.20(s), 3.50 (dd), 4.03(t)	$N\text{-}(CH_3)_3$, $N\text{-}CH_2$, CH_2OH
23	Methanol	3.34(s)	CH_3
24	Carnitine	3.21(s)	CH_3
25	Sn-Glycero-3-phosphocholine	3.23(s), 3.60(dd), 3.68(dd), 3.87(m), 3.94(m), 4.33(m)	$N\text{-}(CH_3)_3$, half $^{1}CH_2$, $^{2}CH_2$, half $^{1}CH_2$, half $^{3}CH_2$, half $^{3}CH_2$, $^{1}CH_2$
26	Taurine	3.24(t), 3.41(t)	$^{1}CH_2$, $^{2}CH_2$
27	Myo-Inositol	3.28(t), 3.53 (dd), 3.63(t), 4.07(t)	^{2}CH, $^{4,6}CH$, $^{1,3}CH$, ^{5}CH
28	Glycine	3.57(s)	$\alpha\text{-}CH_2$
29	Threonine	1.31(d), 3.59(d), 4.25(m)	$\gamma\text{-}CH_2$, β-CH
30	Lactate	1.33(d), 4.11(q)	$\beta\text{-}CH_3$, α-CH
31	NAD^+	6.03(d), 6.08(s), 8.16(s), 8.20(m), 8.41(s), 8.82(d), 9.13(d), 9.32(s)	NH_2, $NH_2(CO)$, δ-CH, β-CH, ^{2}CH, γ-CH, α-CH
32	AXP	6.14 (d), 8.27 (s), 8.58 (s)	NH_2, δ-CH, ^{2}CH
33	Glucose	β(3.24 (dd), 3.48(t), 3.90 (dd)), α(3.54 (dd), 3.71(t), 3.72 (dd), 3.83(m))	β(H2, H3, H5), α(H2, H3, H6)
34	Fumarate	8.46(s)	CH
35	Histidine	7.06(s), 7.85(s)	$^{5}CH_2$, ^{2}CH
36	Tyrosine	3.05(dd), 3.19(dd), 6.92(d), 7.19(d)	half $\beta\text{-}CH_2$, half $\beta\text{-}CH_2$, β-CH, α-CH
37	Phenylalanine	3.12(dd), 3.30(dd), 3.99(dd), 7.33(d), 7.37(t), 7.43(t)	α-CH, half $\beta\text{-}CH_2$, half $\beta\text{-}CH_2$, α-CH, β-CH, γ-CH

Table S2. Relative levels of metabolites calculated from 1D ^{1}H-NMR spectra of aqueous metabolites extracted from the two groups of C2C12 myoblasts

Metabolites	Mean ± SD		Lac vs. Con	*t*-test	
	Control	Lactate		F	p
Pantothenate	0.117±0.098	0.067±0.008	NS	4.374	0.177
Leucine	0.816±0.077	0.784±0.033	NS	1.536	0.304
Isoleucine	0.376±0.020	0.364±0.015	NS	0.896	0.193
Valine	0.388±0.014	0.385±0.016	NS	0.840	0.647
Ethanol	0.056±0.012	0.067±0.014	NS	0.066	0.122
Hydroxybutyrate	0.025±0.015	0.042±0.025	NS	2.668	0.115
Alanine	4.065±0.120	4.418±0.086	↑↑↑	2.108	0.000
Acetate	0.176±0.038	0.151±0.035	NS	0.066	0.190
Proline	0.622±0.047	0.643±0.037	NS	0.623	0.332
Glutamate	3.637±0.086	3.764±0.102	↑	0.171	0.017
Succinate	0.181±0.013	0.163±0.008	↓↓	2.015	0.004
Glutamine	1.468±0.085	1.623±0.047	↑↑↑	4.037	0.000
Methionine	0.109±0.005	0.113±0.003	NS	6.751	0.147
Aspartate	0.060±0.013	0.051±0.014	NS	0.281	0.203
Asparagine	0.019±0.007	0.018±0.008	NS	0.254	0.760
Trimethylamine	0.004±0.002	0.005±0.002	NS	0.077	0.681

Glutathione	0.578±0.044	0.615±0.023	NS	2.563	0.057
Lysine	0.092±0.009	0.097±0.006	NS	1.066	0.222
Creatine	1.635±0.099	1.541±0.125	NS	0.455	0.120
Phosphocreatine	1.052±0.088	1.186±0.101	↑	0.004	0.013
carnitine	1.634±0.099	1.541±0.125	NS	0.455	0.120
β-Alanine	0.440±0.020	0.452±0.012	NS	1.998	0.186
Choline	0.096±0.010	0.103±0.006	NS	2.059	0.082
Sn-Glycero-3-phosphocholine	1.231±0.103	1.443±0.048	↑↑↑	4.732	0.000
Methanol	0.980±0.164	1.316±0.597	NS	4.340	0.147
Taurine	2.387±0.063	2.431±0.044	NS	1.951	0.133
MyoInositol	2.921±0.114	3.447±0.069	↑↑↑	1.755	0.000
Glycine	2.878±0.048	3.032±0.061	↑↑↑	0.970	0.000
Threonine	0.221±0.020	0.209±0.001	NS	8.299	0.141
Lactate	1.751±0.240	1.938±0.068	NS	6.297	0.066
NAD^+	0.035±0.003	0.039±0.003	↑	0.153	0.019
AXP	0.614±0.019	0.598±0.017	NS	0.157	0.089
Glucose	0.079±0.029	0.075±0.014	NS	2.316	0.711
Fumarate	0.010±0.002	0.009±0.001	NS	1.028	0.600
Methylhistidine	0.017±0.001	0.018±0.001	NS	0.079	0.107
Tyrosine	0.177±0.007	0.179±0.005	NS	1.103	0.470
Phenylalanine	0.134±0.006	0.137±0.004	NS	1.895	0.250

PS: ↑/↓: $p < 0.05$, ↑↑/↓↓: $p < 0.01$, ↑↑↑/↓↓↓: $p < 0.001$, Red and blue represent upregulation and downregulation, respectively.

Table S3. Significantly altered metabolic pathways in lactate-treated C2C12 myoblasts relative to controls.

NO	Metabolic Pathway	*P*-values	Impact values
1	alanine, aspartate and glutamate metabolism	0.000449	0.3109
2	D-Glutamine and D-glutamate metabolism	0.000471	0.5
3	glyoxylate and dicarboxylate metabolism	0.000672	0.10582
4	arginine biosynthesis	0.002785	0.11675
5	glutathione metabolism	0.011075	0.10839
